# Electrochemical Properties of Phytosynthesized Gold Nanoparticles for Electrosensing

**DOI:** 10.3390/s22010311

**Published:** 2021-12-31

**Authors:** Natalia Yu. Stozhko, Maria A. Bukharinova, Ekaterina I. Khamzina, Aleksey V. Tarasov

**Affiliations:** 1 Department of Physics and Chemistry, Ural State University of Economics, 8 Marta St., 62, 620144 Yekaterinburg, Russia; xei260296@mail.ru; 2 Scientific and Innovation Center of Sensor Technologies, Ural State University of Economics, 8 Marta St., 62, 620144 Yekaterinburg, Russia; m.a.buharinova@usue.ru (M.A.B.); tarasov_a.v@bk.ru (A.V.T.)

**Keywords:** green synthesis, phytosynthesis, plant extract, antioxidant activity, gold nanoparticles, electrochemical properties, size effect, electrosensing, uric and ascorbic acids

## Abstract

Gold nanoparticles are widely used in electrosensing. The current trend is to phytosynthesize gold nanoparticles (phyto-AuNPs) on the basis of the “green” chemistry approach. Phyto-AuNPs are biologically and catalytically active, stable and biocompatible, which opens up broad perspectives in a variety of applications, including tactile, wearable (bio)sensors. However, the electrochemistry of phytosynthesized nanoparticles is not sufficiently studied. This work offers a comprehensive study of the electrochemical activity of phyto-AuNPs depending on the synthesis conditions. It was found that with an increase in the aliquot of the plant extract, its antioxidant activity (AOA) and pH, the electrochemical activity of phyto-AuNPs grows, which is reflected in the peak potential decrease and an increase in the peak current of phyto-AuNPs electrooxidation. It has been shown that AOA is an important parameter for obtaining phyto-AuNPs with desired properties. Electrodes modified with phyto-AuNPs have demonstrated better analytical characteristics than electrodes with citrate AuNPs in detecting uric and ascorbic acids under model conditions. The data about the phyto-AuNPs’ electrochemistry may be useful for creating highly effective epidermal sensors with good biocompatibility.

## 1. Introduction

Gold nanoparticles are widely used in various fields of science and technology, including electronics, nanoengineering, sensorics, biotechnology, medicine, cosmetology, and pharmacy. Gold nanoparticles stand out due to their unique optical, physical, and electrochemical properties [1]. A variety of physical and chemical methods are used to obtain gold nanoparticles. Compared with physical methods of synthesis, chemical ones are quite simple, labor-saving, and inexpensive. However, reagents and solvents used in chemical synthesis, as well as reaction byproducts, can have a harmful impact on the human body and the environment [2,3]. Recently, alternative methods of nanoparticle synthesis, based on the “green” chemistry approach, have emerged. “Green” synthesis employs plant extracts (phytosynthesis) that serve as reducing, stabilizing, and capping agents. The “green” method is an effective eco-friendly methodology for obtaining gold nanoparticles [4]. The main benefits of phytosynthesis include environmental safety, simplicity and high synthesis rate, no need for additional reagents, low cost, and the possibility of large-scale production of nanoparticles [4,5,6].

The distinctive features of phytosynthesized gold nanoparticles (phyto-AuNPs) are their high catalytic ability in the process of organic dye degradation [2,7]; antibacterial [8], anticancer [9], and antioxidant activity [8,9,10]; and biocompatibility and low cytotoxicity [5], which makes phyto-AuNPs very attractive for biomedical applications, including theranostics, diagnostic studies, cell imaging, photodynamic therapy, as well as drug, protein, and gene delivery [5,11]. In electrosensing, phyto-AuNPs have not been frequently used yet, which is primarily due to the lack of knowledge about their electrochemistry. A few studies have been published that describe phyto-AuNPs as electrode modifiers. The use of electrodes based on “green” gold nanoparticles helps to determine chloramphenicol in milk, honey, and eye drops [12]; nitrite ions in tap and mineral water [13]; carbendazim in soil [14]; lead ions in paints and river waters [15]; heavy metal ions [16]; hydrazine [17], hydroquinone, catechin, and resorcinol [18]; and ascorbic acid in juices [19]. A comparative study has shown that the phyto-AuNPs sensor has a higher sensitivity, better stability, and reproducibility of the electrochemical response to the analyte concentration in comparison with an electrode modified with gold nanoparticles synthesized by the traditional Turkevich method [19]. This fact contributes to the prospects of using phyto-AuNPs in electrosensing.

The most recent development of skin-attachable (epidermal) sensors [20,21,22,23], including those with electrochemical methods of signal detection, suggests a rapid transfer of these devices from a research laboratory to a clinical environment for health monitoring and disease diagnosis. Biocompatibility of such devices plays an important role in ensuring their safe interaction with living cells of the epidermis, which can be significantly improved as a result of applying “green” nanomaterials obtained using medicinal and food plants. This determines the relevance of the current research that is aimed at studying electrochemical properties of biocompatible phytosynthesized gold nanoparticles for further application in epidermal electrosensing.

It is commonly known that the electrochemical properties of nanoparticles are size-dependent [24]. This is demonstrated both in the transformation process of metal nanoparticles, and in the electrical transformation of the substance on the nanoparticle surface [25]. The findings show that the size of nanoparticles is affected by the conditions of “green synthesis”, such as concentration and volume of the precursor; aliquot of plant extract; the temperature of synthesis; and pH of the reaction mixture [26,27,28]. Thus, a higher temperature results in the formation of smaller nanoparticles and higher formation rate [29]. Another factor that affects the kinetics of synthesis, the size of the resulting phytonanoparticles, and their stability is the antioxidant activity (AOA) of the plant extract and the reaction mixture during phytosynthesis [30]. It has been shown that with an increase in the AOA of the plant extract, the rate of phytosynthesis increases; phyto-AuNPs become smaller and the proportion of small phyto-AuNPs d ≤ 5 nm increases, while the proportion of large phyto-AuNPs d ≥ 31–50 nm decreases. The stability of phytosols also grows.

The changes in the size and the share of nanoparticles of a certain size during phytosynthesis cannot but affect both their own electrochemical properties and the properties of substances that undergo electrical conversion on nanoparticles. In this regard, the study of the electrochemistry of phytosynthesized nanoparticles in terms of the impact of phytosynthesis conditions is a challenging research and practical task, since its solution will enable to control and monitor the process of phytosynthesis and obtain nanoparticles with the desired properties.

Thus, the aim of the paper is to study the electrochemical properties of gold nanoparticles against the variable parameters of phytosynthesis, including different aliquots, AOA and pH of plant extracts from strawberry, black currant, and gooseberry leaves and to compare analytical characteristics of electrodes modified with phyto-AuNPs and AuNPs synthesized with the traditional Turkevich method.

## 2. Materials and Methods

### 2.1. Chemicals and Reagents

The following chemicals were used: Na_2_HPO_4_·12H_2_O (JSC Vekton, St. Petersburg, Russia); KH_2_PO_4_ (NevaReaktiv Ltd., St. Petersburg, Russia), K_3_[Fe(CN)_6_)] and K_4_[Fe(CN)_6_]·3H_2_O (AO Reachim Ltd., Moscow, Russia); KCl (JSC ChemReactivSnab, Ufa, Russia), Na_3_C_6_H_5_O_7_·5.5H_2_O (JSC ChemReactivSnab, Ufa, Russia), HAuCl_4_ (RPE Tom’analit Ltd., Tomsk, Russia), HCl (NevaReactiv Ltd., St. Petersburg, Russia), and NaOH (JSC ChemReactivSnab, Ufa, Russia). All chemicals were used without extra purification. The solvent was deionized water (resistivity of 18 MΩ cm).

### 2.2. Equipment and Electrodes

Dispersiveness of plant powders was ensured with a sieve (mesh size: 0.08 mm) (Kraft Ltd., Yekaterinburg, Russia). A magnetic stirrer with controlled heating RCT Basic (IKA—Werke GmbH & Co. KG, Staufen, Germany) and a MIKRO 120 centrifuge (Andreas Hettich GmbH, Tuttlingen, Germany) were used for the preparation of plant leaf extracts, synthesis, and purification of phyto-AuNPs. AOA of the plant extracts was measured by a multifunctional potentiometric analyzer MPA-1 (IVA Ltd., Yekaterinburg, Russia) with the use of a two-electrode cell composed of a platinum screen-printed electrode (IVA Ltd., Yekaterinburg, Russia) and a silver chloride electrode (Ag/AgCl/KCl, 3.5 M EVL-1M3.1 type) (JSC Gomel Plant of Measuring Devices, Gomel, Belarus) as the indicator and reference electrodes, respectively. The pH of the plant extracts was determined using pH/ions-meter TA-Ion (RPE Tomanalit Ltd., Tomsk, Russia).

Phyto-AuNPs characterization was carried out with UV–visible (UV–vis) spectrophotometry, dynamic light scattering (DLS), electrochemical impedance spectroscopy (EIS), linear sweep voltammetry (LSV) and cyclic voltammetry (CV). UV–vis spectrophotometric measurements were taken with ECO-VIEW UV 1200 spectrophotometer (Shanghai Mapada Instruments Co., Ltd., Shanghai, China) by using standard 10 mm quartz cuvettes. DLS measurements were performed on a BrookHaven ZetaPlus analyzer (Brookhaven Instruments Corp., Holtsville, NY, USA). EIS measurements were carried out using a µAutolab Type III potentiostat/galvanostat (Metrohm, Herisau, Switzerland). LSV and CV measurements were made with an voltammetric analyzer IVA-5 (IVA Ltd., Yekaterinburg, Russia) using a three-electrode cell, composed of an empty or modified working electrode; a silver chloride electrode (Ag/AgCl/KCl, 3.5 M EVL-1M3.1 type) (JSC Gomel Plant of Measuring Devices, Gomel, Belarus) as the reference electrode; and a glass carbon rod 3 mm/100 mm, GC–2000 type (JSC NIIGrafit, Moscow, Russia) as the auxiliary electrode. A glass carbon electrode (GCE) in a polyaryletheretherketone case (disk d = 2 mm) (Metrohm AG, Switzerland); an electrode based on Ceres carbon ink (Guangzhou Print Area Technology Co., Ltd., Foshan, China) and PET (SPE), manufactured by screen printing using a SPR-10 manual printer (DDM Novastar Inc., Warminster, PA, USA) and a polyamide mesh stencil, were used as empty working electrodes. The applied carbon ink was baked in the oven at 110 °C; for 30 min. Modification of GCE or SPE with phyto-AuNPs was performed as follows. An amount of 10 µL of phyto-AuNPs sol was applied drop-by-drop onto the electrode surface; then, the electrode was dried at ambient temperature.

Akvalab-UVOI-MF-1812 installation (JSC RPC Mediana-Filter, Moscow, Russia) was used to obtain deionized water with a resistivity of 18 MΩ cm.

### 2.3. Procedures

#### 2.3.1. Plant Extracts Preparation

Aqueous extracts from strawberry (*Fragaria vesca*), black currant (*Ribes nigrum*), and gooseberry (*Ribes uva-crispa*) leaves were obtained by hydrothermal extraction [30,31]. Plant leaves were dried at room temperature, crushed in a mortar, and sieved through a sieve with 0.08 mm mesh size. A suspension of freshly prepared powder weighing 0.4 g was placed in a heat-resistant flask filled with 10 mL of deionized water. Extraction was carried out at a temperature of 80 °C for 20 min with constant stirring. Then, the obtained suspension was cooled to room temperature. The liquid and solid particles of the suspension were separated by centrifugation at 10,000 rpm for 5 min. For each consequent analysis, freshly prepared plant extracts were used. pH 3 and pH 12 of the extracts were obtained using 1 M HCl and 5 M NaOH, respectively. To obtain the pH 3 value, 8–10 µL of 1 M HCl was micropipetted onto 9.5 mL of the plant extract (pH 6). To obtain the pH 12 value, 3–5 µL of 5 M NaOH was added to 9.5 mL of the initial plant extract (pH 6). Since the volume of HCl and NaOH did not exceed 0.01 mL, the dilution of the plant extract was insignificant and it was not taken into account.

#### 2.3.2. AOA Determination

The AOA of freshly prepared plant extracts with different pH values was measured with the use of the potentiometric method and the two-electrode cell in accordance with [31]. The platinum screen-printed electrode was pre-cleaned by high-temperature annealing. Summarily, 0.2 mL of plant extract (pH 6) was added to 9.8 mL of pH 7 phosphate buffer containing 10 mM K_3_[Fe(CN)_6_] and 0.1 mM K_4_[Fe(CN)_6_]. AOA of extracts with pH 3 and pH 12 was measured in a similar way.

#### 2.3.3. Synthesis of Gold Nanoparticles

In order to produce gold sols, different volumes of plant extract (V_extr_ = 0.25–1.0 mL) were mixed with 5.0 mL of boiling and intensively stirred precursor solution (1 mM HAuCl_4_). The first indication of the nanoparticle formation was the change of the color of the reaction mixture. After 5 min the phytosynthesis was stopped. A freshly synthesized phyto-AuNPs sol was cooled to ambient temperature and washed. For this purpose, 1.0 mL of phyto-AuNPs sol was centrifuged for 10 min at 14,000 rpm. Then, the supernatant was taken out and the precipitate was resuspended in the same amount of deionized water. The procedure was repeated twice. After washing, the phyto-AuNPs sol was resuspended with deionized water to an initial volume (1.0 mL). The prepared phyto-AuNPs sol was used for electrochemical studies and stored at a temperature of 4 °C. Gold nanoparticles obtained with the use of extracts from strawberry, black currant, and gooseberry leaves were marked as sb-AuNPs, bc-AuNPs, and gb-AuNPs, respectively. When studying the effect of the plant extract pH, 0.75 mL of strawberry leaf extract or 1.0 mL of the extracts of black currant and gooseberry leaves with different pH values were added to 5.0 mL of the boiling precursor. The plant extract pH values were varied using 1 M HCl and 5 M NaOH solutions.

Citrate gold nanoparticles (cit-AuNPs) were synthesized with the help of the Turkevich method [32]. Briefly, 750 µL of 0.1 M sodium citrate was added to 15 mL of a boiling 1 mM HAuCl_4_ solution with constant stirring.

#### 2.3.4. Characterization of Phyto-AuNPs

In the experiments with the use of UV–vis spectrophotometry and DLS, phyto-AuNPs sol samples were diluted three times with deionized water, since the intense color of the sols hindered measurements. Optical spectra of phytosols were recorded in the wavelength range of 450 to 650 nm. Deionized water was used as a blank matrix.

While analyzing phyto-AuNPs sols with the use of DLS, 5.0 mL of the sample was placed in a plastic transparent cuvette, and the autocorrelation function was obtained using the operating mode of the particle sizing software device. In order to measure zeta potential, 2.5 mL of the sample was placed in the cuvette and electrochemical parameters were recorded with the Zeta Potential Analyzer. Information about the shape and size of phyto-AuNPs obtained with transmission electron microscopy (TEM) was also added from our earlier work [30].

#### 2.3.5. Electrochemical Measurements

Electrochemical measurements were performed comparing a phyto-AuNPs/GCE with an empty GCE. Prior to measurements, the electrodes were prepared in accordance with the operating manual. GCE were polished on a polishing cloth and rinsed with double distilled water. SPE, phyto-AuNPs/GCE, and phyto-AuNPs/SPE were only rinsed with double distilled water. Linear sweep (LS) voltammograms of phyto-AuNPs electro-oxidation were recorded in 1 M HCl in the potential range from 0.6 V to 1.1 V at a sweep rate of 0.05 Vs^−1^. LS of uric acid electro-oxidation were registered in pH 5 phosphate buffer solution (PBS) in the range from 0.20 to 0.85 V at potential scan rate of 0.05 Vs^−1^. Cyclic voltammograms were recorded in a solution containing 5.0 mM K_3_[Fe(CN)_6_], 5.0 mM K_4_[Fe(CN)_6_], and 0.1 M KCl, in the potential range from −0.3 V to 1.1 V at a sweep rate of 0.05 Vs^−1^. The received voltammograms were characterized in accordance with [33]. EIS measurements were carried out in supporting 0.1 M KCl in the presence of 1.0 mM [Fe(CN)_6_]^3−/4−^. The frequency range was from 0.1 Hz to 100 kHz with amplitude 5 mV at a polarization potential of 0.25 V.

### 2.4. Data Treatment

The AOA of the plant extract was calculated as follows Equation (1):

(1) $$AOA=\frac{C_{Ox\;}-\alpha C_{Red}}{1+\alpha }\cdot b,\;\alpha =\frac{C_{Ox}}{C_{Red}}\cdot 10^{\Delta EF/2.3RT}$$

where *C_Ox_*—concentration of oxidized component of the mediator system (K_3_[Fe(CN)_6_]), M; *C_Red_*—concentration of reduced component of the mediator system, (K_4_[Fe(CN)_6_]), M; *b*—dilution factor of the sample in the cell; Δ*E*—potential difference of the mediator system after the introduction of the analyzed sample, V; *F* = 96,485.332 C mol^−1^—the Faraday constant; *R* = 8.314 J mol^−1^K^−1^—universal gas constant; and *T*—temperature, K.

The diameter of phyto-AuNPs from the UV–vis spectra data was calculated as expressed by Equation (2) in Haiss et al. [34]:

(2) $$d=e^{\left(B_{1}\frac{A_{SPR}}{A_{450}}-B_{2}\right)}$$

where *d*—the diameter of the phyto-AuNPs, nm; *A_SPR_* and *A*_450_—the absorbance at the surface plasmon resonance peak and 450 nm, a.u.; and *B*_1_ = 3.55, *B*_2_ = 3.11—fit parameters [34].

**Figure 1:**
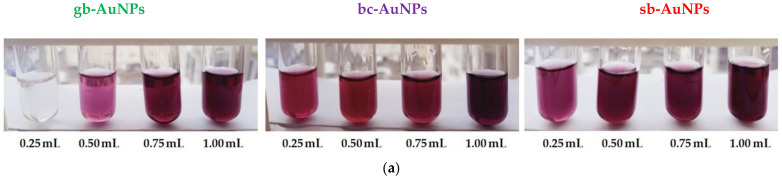

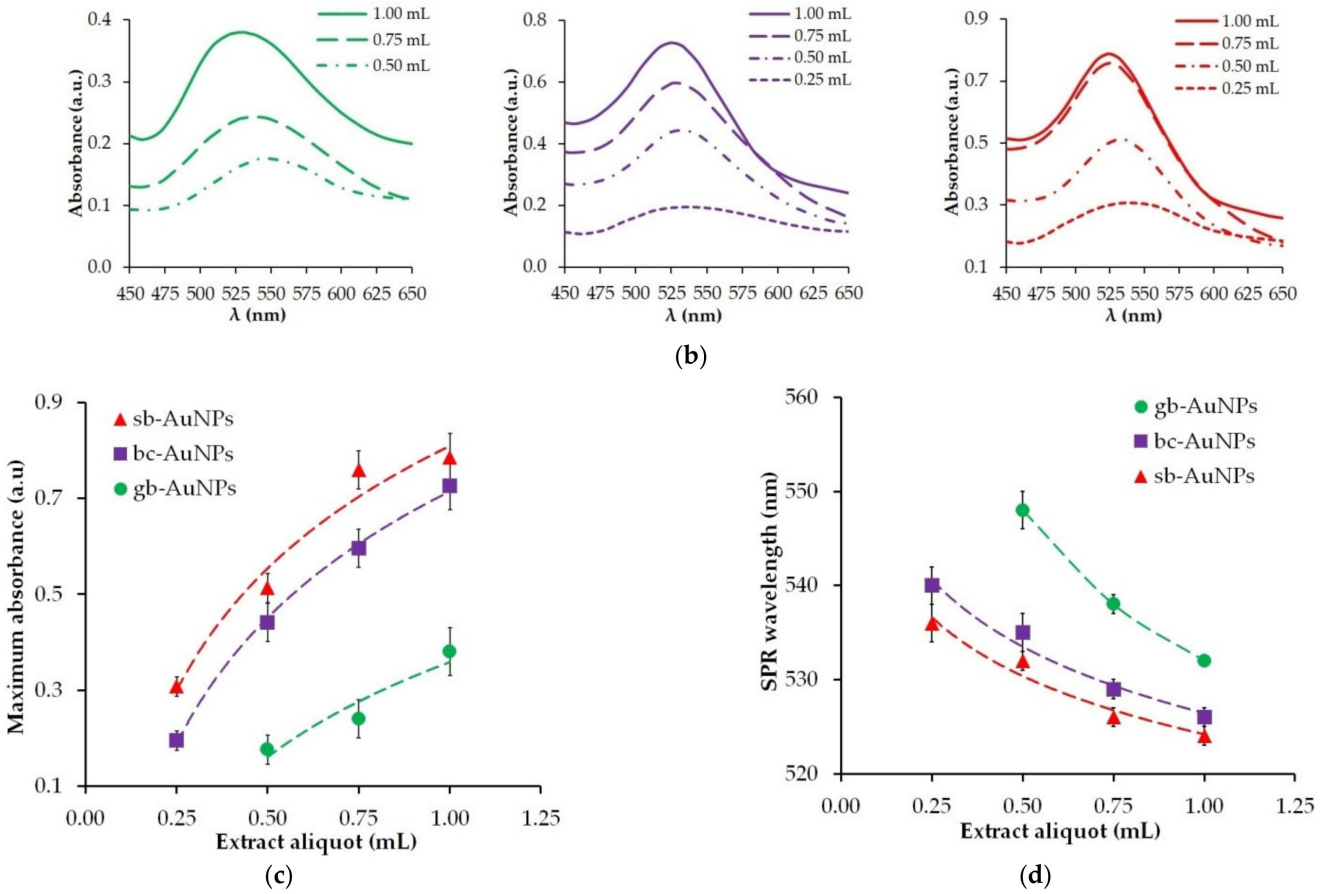
Images of gold sols synthesized with plant extract aliquots (**a**) and corresponding UV–vis spectra (**b**). The impact of extract aliquot (pH 6) on maximum absorbance (**c**) and the position of the characteristic SPR band (**d**) of phyto-AuNPs.

The concentration of synthesized phyto-AuNPs was calculated as expressed by Equation (3) [34] using registered UV–vis spectra (Figure 1):

(3) $$N=\frac{A_{450}\times 10^{14}}{d^{2}\left[-0.295+1.36exp\left(-{\left(\frac{d-96.8}{78.2}\right)}^{2}\right)\right]}$$

Measurements of each parameter were repeated three times minimum with an accepted significance level of 0.05. The data obtained are presented as a mean value ± standard deviation.

## 3. Results

Gold nanoparticles synthesized with strawberry, black currant, and gooseberry leaf extracts have a predominantly spherical shape. Their size is affected by an aliquot and AOA of the plant extract, which is confirmed by the findings of TEM, DLS, and UV–vis-spectrophotometry [30]. Since the electrochemical behavior of nanoparticles is determined primarily by their size [24,35], the present experiment was aimed at studying electrochemical activity of gold nanoparticles synthesized with the use of various aliquots, AOA, and pH of strawberry, black currant, and gooseberry leaf extracts. The experiment also focused on studying the process of electrotransformation of K_3_[Fe(CN)_6_]/K_4_[Fe(CN)_6_] on the surface of phytonanoparticles. The electrochemical activity of phyto-AuNPs was evaluated by the peak potential and the peak current of gold nanoparticle oxidation.

### 3.1. The Impact of Washing

The composition of plant extracts is complex and diverse. It includes polyphenolic compounds, flavonoids, enzymes, amino acids, polysaccharides, proteins, alkaloids, tannins, organic acids, saponins, ketones, and aldehydes [36,37]. During phytosynthesis, these compounds act as bioreducing, capping, and stabilizing substances that form a phytofunctional coating on the surface of nanoparticles. This coating protects nanoparticles from aggregation [38]. In phytosynthesis, not all biomolecules of plant extracts can interact with Au(III) ions to form nanoparticles; some of them may stay in the solution, especially if large aliquots and/or high concentrations of plant extracts are used. If the electrode is modified with such a sol, then it can be expected that the unreacted biomolecules may firmly adsorb on the electrode surface and affect the electrode process. In order to find out whether the excess of unreacted plant extract impacts the electrochemical activity of phyto-AuNPs, we compared the electrochemical properties of the initial gold sols and the gold sols washed from the excess extract that were synthesized with the use of a large aliquot (0.75 mL) of plant extracts. The results of this experiment are presented in Table 1.

As can be seen from Table 1, the peak potential of washed sb-AuNPs electro-oxidation has not changed in comparison with unwashed sb-AuNPs, while for bc-AuNPs and gb-AuNPs, the peak potential has grown by 10 mV and 50 mV, respectively. At the same time, the peak current of washed phyto-AuNPs electro-oxidation has increased by more than four times in comparison with unwashed phyto-AuNPs for all plant extracts. The results obtained show that after purification phyto-AuNPs become more electrochemically active compared with non-purified phyto-AuNPs. Further studies were carried out using washed phyto-AuNPs only.

### 3.2. The Impact of Aliquots

The optical properties of gold sols are characterized by a surface plasmon resonance (SPR) band at 525–560 nm, resulting from collective oscillations of free conduction electrons when excited by an external electromagnetic field. The maximum absorption and wavelength of the characteristic SPR band in the UV–visible spectrum are sensitive to the content of colloidal gold nanoparticles and their size. The shift of the SPR wavelength to the short-wavelength area indicates a decrease in the size of nanoparticles in gold sols [39]. The images of phytosynthesized gold sols and the change in maximum absorption and SPR wavelength resulting from aliquots of strawberry, black currant, and gooseberry extracts are presented in Figure 1. During phytosynthesis, burgundy gold sols are formed, and their color intensity grows with an increase in the aliquot of the extract (Figure 1a). Only in one case phyto-AuNPs are not formed: when the smallest aliquot (0.25 mL) of the gooseberry leaf extract is used and it has the lowest AOA compared with other plants. As illustrated by Figure 1c,d, the highest maximum absorption and shortest SPR wavelength are observed for sb-AuNPs, and the lowest maximum absorption and longest SPR wavelength—for gb-AuNPs. With an increase in the aliquot of plant extracts, there is an increase in maximum absorption (Figure 1c) and a decrease in SPR wavelength (Figure 1d). 

Based on the UV–vis spectrophotometer data (Figure 1b), the sizes and concentrations of phyto-AuNPs in the sols, presented in Table S1 in the Supplementary Materials, were calculated. There is a trend toward a smaller size and higher concentration of nanoparticles when the aliquot of the plant extract (pH 6) used in phytosynthesis becomes larger. When using the same aliquot of extracts of different plants, a similar trend is observed in the gb-AuNPs–bc-AuNPs–sb-AuNPs series. Consequently, the largest number of the smallest gold nanoparticles is formed when 1.0 mL of strawberry leaf extract is used.

**Figure 2:**
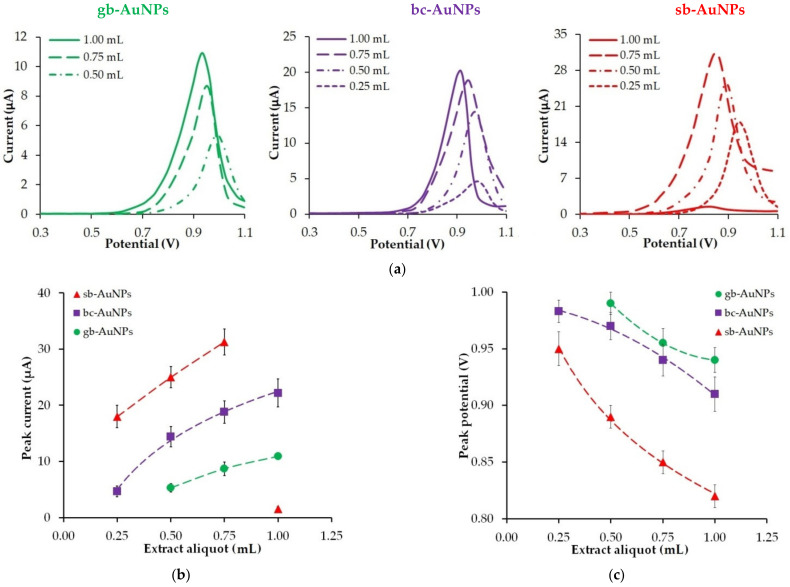
LS voltammograms of phyto-AuNPs electrooxidation (**a**). Background: 1 M HCl, potential scan rate 0.05 Vs^−1^. The impact of extract aliquots (pH 6) used in phytosynthesis on the peak current (**b**) and the peak potential (**c**) of phyto-AuNPs electro-oxidation. Synthesis conditions of phyto-AuNPs: C(HAuCl_4_) = 1 mM, V_extr_ = 0.25−1.00 mL, and pH 6 all of extracts.

A decrease in the size of phyto-AuNPs along with an increase in the aliquot of plant extracts affects the electrochemical properties of phytosynthesized gold nanoparticles (Figure 2). Thus, an increase in the volume of neutral plant extract (pH 6) leads to an increase in the peak oxidation current (Figure 2b) of phyto-AuNPs and its shift to the cathode region (Figure 2c), which indicates higher electrochemical activity of gold nanoparticles with a bigger extract aliquot. If the aliquot of different plant extracts is the same, the following patterns can be observed: there is an increase in the peak current and a decrease in the peak potential of gold nanoparticle oxidation in the “gooseberry—black currant—strawberry” series. The exception is nanoparticles synthesized with the use of 1.0 mL of strawberry leaf extract. In this case, there is a sharp drop in the peak current of the sb-AuNPs oxidation (Figure 2b).

**Figure 3:**
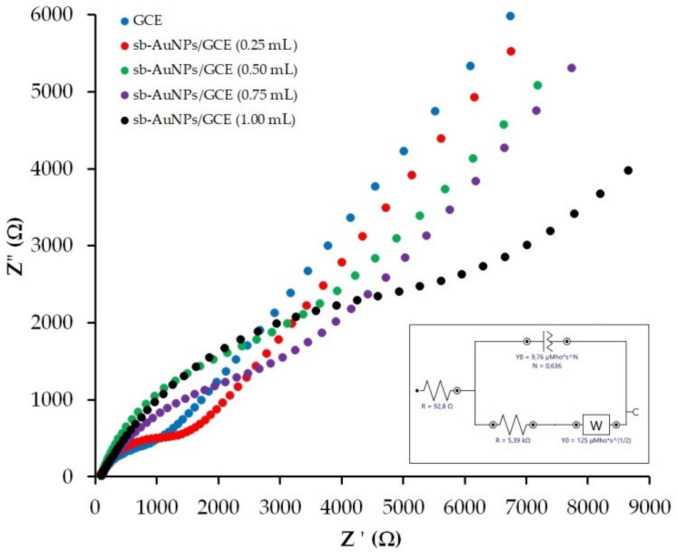
Nyquist plots for GCE and sb-AuNPs/GCE in the presence of 1.0 mM [Fe(CN)6]^3−/4−^ in supporting 0.1 M KCl. Synthesis conditions of sb-AuNPs: C(HAuCl_4_) = 1 mM, V_extr_ = 0.25−1.0 mL, and pH_extr_ 6. Frequency range 0.1 Hz–100 kHz at polarization potential of 0.25 V and amplitude of 5 mV. Inset: the diagram of a Randles equivalent cell.

In order to understand why the peak current of sb-AuNPs electro-oxidation falls sharply at a 1 mL aliquot, the electron transport properties of an empty GCE and sb-AuNPs/GCEs were studied with the help of the EIS method. Figure 3 represents the experimental Nyquist plots of the GCE and sb-AuNPs/GCEs. Table 2 demonstrates the results of the EIS spectra fitting using the Randles equivalent circuit. As can be seen from the presented data, modification of the GCE with sb-AuNPs leads to an increase in the semicircle diameter and indicates growth in the interfacial electron-transfer resistance in comparison with the GCE. The larger the aliquot of the neutral extract (pH 6) of strawberry leaves used in the synthesis of gold sols, the larger the semicircle diameter. The highest electron transfer resistance is observed on sb-AuNPs synthesized using a 1.0 mL aliquot. Thus, sb-AuNPs/GCE (1.0 mL) is characterized by almost 10-fold greater charge transfer resistance compared with the GCE (8.4 ± 1.2 vs. 0.87 ± 0.11 kΩ for the GCE) (Table 2), which confirms the lowest electron transfer rate among the discussed sb-AuNPs/GCEs.

Two assumptions were made with regard to the observed phenomenon. An extremely high charge transfer resistance for sb-AuNPs/GCE (1.0 mL) may be explained either by the blocking effect of an excessive unreacted plant extract, or by the formation of a blocking coating on the nanoparticle itself. To test the first assumption, the gold sol synthesized with 1.0 mL of the strawberry leaf extract was washed three, four, and five times. If this assumption were correct, it could be expected that the current of gold nanoparticle electro-oxidation would increase with a higher number of washings. However, the obtained results did not justify this assumption. The peak current of sb-AuNPs electro-oxidation not only increased, but, on the contrary, decreased by 17%, 55%, and 57% for nanoparticles washed three, four, and five times, respectively, as compared with nanoparticles washed only twice. Moreover, the color of the gold sol changed with each washing: from burgundy (two washings), to deep purple (three washings), and pale purple (four and five washings), which indicates the enlargement of nanoparticles and a change in their concentration in the sol. After four and five washings, a dark clot of aggregated nanoparticles was observed at the bottom of the vessel. In accordance with the results of this experiment, it was concluded that the effect of the electrode blocking is due to the formation of a “capping” (blocking) shell on the nanoparticle in large aliquots of pH 6 strawberry leaf extract rather than to an excess of the unreacted extract. This shell erects the energy barrier of electron transfer between the electrode and gold nanoparticles [40]. Based on the results of the studies [41,42] that demonstrated the electron tunneling on isolated gold [42] and silver nanoparticles [41], it can be assumed that in our case, the use of large aliquots of the neutral strawberry leaf extract (pH 6) results in a higher Coulomb potential barrier and the blocking of the electron tunneling, i.e., the so-called Coulomb blockade can be observed.

Nevertheless, the obtained results show that in the presence of neutral strawberry leaf extract (no more than 0.75 mL), the most electrochemically active—in comparison with black currant and gooseberry leaf extracts—phyto-AuNPs are formed. This is primarily due to the fact that the size of nanoparticles decreases in the gb-AuNPs–bc-AuNPs–sb-AuNPs series, which is confirmed by the above-mentioned results of optical studies and earlier obtained data [30]. The diameter of phyto-AuNPs formed with gooseberry, black currant, and strawberry leaf extracts is 25 nm, 11 nm, and 10 nm, respectively, according to UV–vis spectrophotometry results; 23 nm, 15 nm, and 14 nm according to TEM results; and 42 nm, 38 nm, and 30 nm according to DLS results [30].

### 3.3. The Impact of Extract AOA

In our earlier study, we found that the AOA of a plant extract significantly affects the kinetics of phytosynthesis and the properties of synthesized phyto-AuNPs [30]. It was shown that with an increase in the AOA of a plant extract the rate of photosynthesis increases, the size of phyto-AuNPs decreases, and their stability increases. It was of interest to find out whether there is a relationship between the AOA of the reaction mixture and the electrochemical properties of phyto-AuNPs. Data on the AOA of aqueous solutions containing different aliquots of plant extracts are presented in Table S2 in the Supplementary Materials. As can be seen from Table S2, an increase in the aliquot of the plant extract (pH 6) causes an increase in the AOA of the solution. It should be noted that with the same aliquot of a plant extract, the AOA of the reaction mixture increases in the “gooseberry > black currant > strawberry” series.

**Figure 4:**
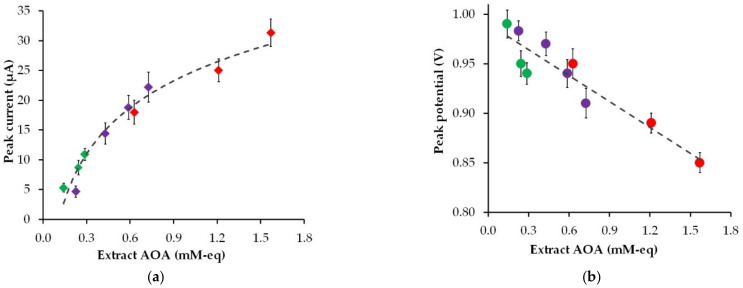
The impact of the AOA of the plant extracts (pH 6) (gooseberry—green marker, black currant—violet marker, and strawberry—red marker) on the peak current (**a**) and the peak potential (**b**) of phyto-AuNPs electro-oxidation.

Figure 4 shows the effect of the AOA of the plant extracts of strawberry, black currant, and gooseberry leaves on the peak current and the peak potential of phyto-synthesized gold nanoparticle electro-oxidation.

As can be found in Figure 4, an increase in the AOA of the plant extract leads to an increase in the peak current and a decrease in the peak potential of phyto-AuNPs electro-oxidation. Moreover, the highest peak current and the lowest peak potential are observed for gold nanoparticles synthesized with strawberry leaf extracts, whose AOA is significantly higher than the AOA of gooseberry and black currant leaf extracts. The visible increase in the electrochemical activity of phyto-AuNPs as a result of an increase in the AOA of plant extracts is due to the dimensional effect, where smaller gold nanoparticles synthesized using phytoextracts with high AOA have greater electrochemical activity than larger particles synthesized using phytoextracts with low AOA.

### 3.4. The Impact of Plant Extract pH

**Figure 5:**
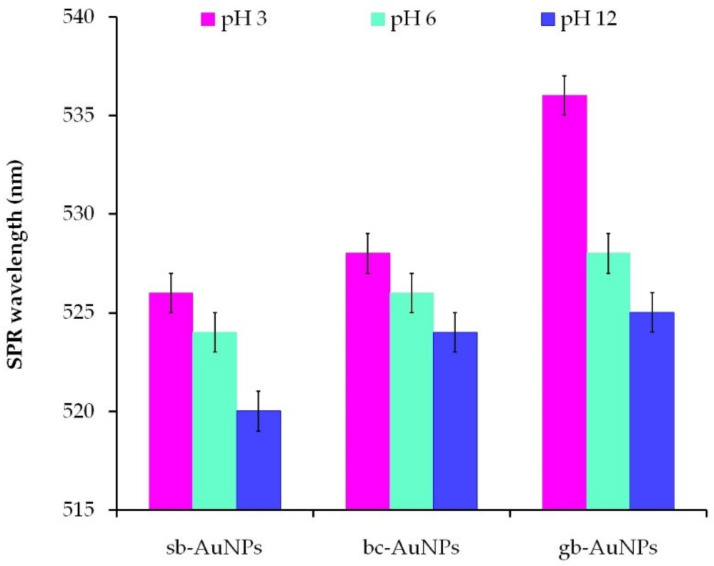
The impact of plant extract pH on the position of the characteristic SPR band of phyto-AuNPs. Synthesis: V_extr_ = 0.75 mL (for sb-AuNPs) and V_extr_ = 1.0 mL (for bc-AuNPs and gb-AuNPs).

To study the effect of the plant extract pH on the properties of phyto-AuNPs, acidic (pH 3), neutral (pH 6), and alkaline (pH 12) plant extracts were used. The AOA of the plant extracts with different pH values is given in Table S2 in the Supplementary Materials. The AOA of strawberry leaf extracts (1 mL aliquot) stays practically the same with a change in the solution pH. For the black currant and gooseberry extracts, it decreases slightly in the alkaline medium as compared with acidic and neutral media. The dependence of the size and stability of phyto-AuNPs on pH of the plant extract was judged by the results of spectrophotometric and DLS studies. As can be seen from Figure 5, an increase in the plant extract pH leads to a shift of the SPR wavelength to the short-wavelength region, which indicates a decrease in the size of phyto-AuNPs. The formation of spherical highly dispersed and ultra-small nanoparticles in the alkaline medium (pH 10–11) was compared against the acidic medium (pH 3–5) and described in [5,43,44,45]. The possible explanation of this phenomenon might be the fact that the rate of nanoparticle formation is higher than the rate of their aggregation in the alkaline medium. The correlation is reversed in the acidic medium: the aggregation of nanoparticles exceeds the nucleation process [44]. It is also worth noting that when pH of the plant extracts changes, the ionic state of their phytocomponents changes too, which can also affect the processes of nucleation, formation, and stabilization of phyto-AuNPs [44,46].

The effect of the extract pH on the stability of phytosynthesized gold sols and the size of nanoparticles was studied using sb-AuNPs synthesized by strawberry leaf extracts with different pH (Table 3). An increase in pH of the strawberry leaf extract from 3 to 12 is accompanied by an increase in the absolute value of the zeta potential, which characterizes the stability of gold sols, and a decrease in the diameter of sb-AuNPs, which was found by DLS and UV–vis spectrophotometry. The formation of more stable gold sols at extracts with high pH was confirmed by R.J.B. Pinto et al. [46]. They suggested [46] that higher pH results in a higher number of anionic biomolecular particles acting as stabilizing agents, which causes an increase in electrostatic repulsion between gold nanoparticles and an increase in their stability.

**Figure 6:**
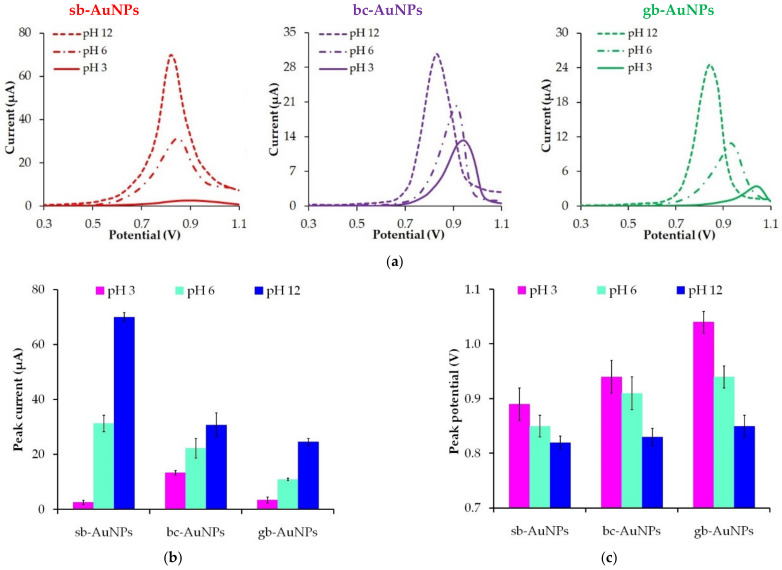
LS voltammograms of phyto-AuNPs electro-oxidation synthesized using plant extracts with different pH (**a**). Background: 1 M HCl, potential scan rate 0.05 Vs^−1^. The impact of the plant extract pH on the peak current (**b**) and the peak potential (**c**) of phyto-AuNPs electro-oxidation. Synthesis conditions: C(HAuCl_4_) = 1 mM, V_extr_ = 0.75 mL (for sb-AuNPs), and V_extr_ = 1.0 mL (for bc-AuNPs and gb-AuNPs).

Figure 6 shows the change in the peak current and the peak potentials of phyto-AuNPs oxidation depending on the pH of strawberry, black currant, and gooseberry leaf extracts. The value of the peak current of phyto-AuNPs electro-oxidation grows when the pH of extracts changes from 3 to 12, while the peak potential falls. Taking into account the results of DLC and UV–vis spectrophotometer studies, it can be argued that smaller and more electrochemically active phyto-AuNPs are formed in the alkaline medium rather than in acidic and neutral media.

**Figure 7:**
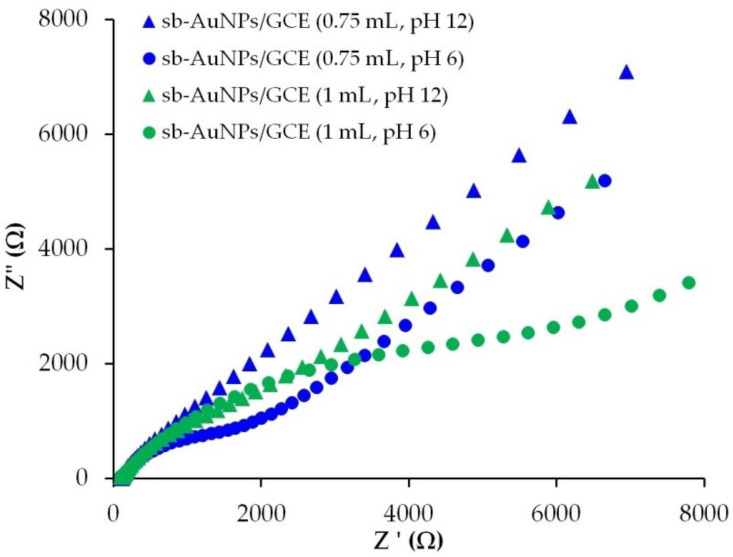
Nyquist plots for sb-AuNPs/GCE in the presence of 1.0 mM [Fe(CN)_6_]^3−/4−^ in supporting 0.1 M KCl. Synthesis conditions of sb-AuNPs: C(HAuCl_4_) = 1 mM, V_extr_ = 0.75, and 1.0 mL, pH_extr_ 6 and 12. Frequency range 0.1 Hz–100 kHz at polarization potential of 0.25 V and amplitude of 5 mV.

Figure 7 illustrates the Nyquist plots for the GCE, modified with sb-AuNPs that are synthesized with 0.75 and 1.0 mL of alkaline (pH 12) and neutral (pH 6) strawberry leaf extracts. As can be seen from Figure 7, the electron transport properties of sb-AuNPs, synthesized with alkaline and neutral plant extracts, differ significantly from each other. For sb-AuNPs/GCE, whose nanoparticles were formed in an alkaline medium, straight lines are observed on the Nyquist plots. The absence of the semicircle for sb-AuNPs, synthesized with both 0.75 and 1 mL of the alkaline strawberry leaf extract, indicates a significant improvement in electron transport properties compared with nanoparticles synthesized in a neutral medium. It can be assumed that OH^−^ ions take an active part in the formation of AuNPs, are embedded in their phyto shell by forming tunnels for electron transfer, and contribute to a lower energy barrier of electron transfer and the removal of the Coulomb blockade for sb-AuNPs (1 mL) [41,42].

### 3.5. Electrochemical Behavior of K_3_[Fe(CN)_6_]/K_4_[Fe(CN)_6_] on Phyto-AuNPs

**Figure 8:**
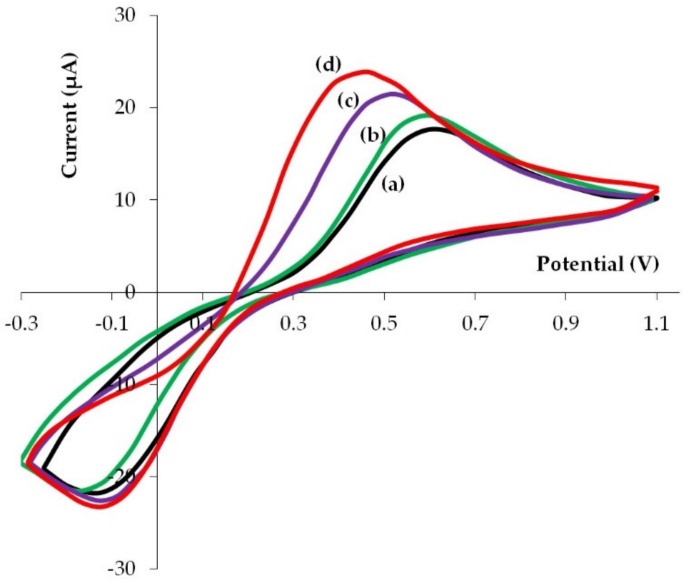
Cyclic voltammograms of 5.0 mM [Fe(CN)_6_]^3−/4−^ in 0.1 M KCl on GCE (**a**) and GCE modified with gold nanoparticles obtained by using 1.0 mL extract of gooseberry (**b**), 1.0 mL extract of black currant (**c**), and 0.75 mL extract of strawberry (**d**) leaves. pH of all extracts: 12. Potential scan rate: 0.05 Vs^−1^.

For a more complete assessment of electrochemical properties of phyto-AuNPs, in addition to the electro-oxidation of gold nanoparticles, the electrochemical transformation of the known reversible system [Fe(CN)_6_]^3−/4−^ on phytonanoparticles was studied. Figure 8 illustrates cyclic voltammograms for [Fe(CN)_6_]^3−/4−^ registered on unmodified electrodes and on electrodes modified with phyto-AuNPs. Cyclic voltammograms clearly show that the potential of the anode peak shifts to the cathode region, and the current peak grows slightly on the modified electrodes in the following sequence: gb-AuNPs/GCE–bc-AuNPs/GCE–sb-AuNPs/GCE. Quantitative indicators of electrochemical transformation of [Fe(CN)_6_]^3−/4−^ on GCE and phyto-AuNPs/GCE are presented in Table 4. In comparison with GCE, lower anode peak potentials (E_a_) of [Fe(CN)_6_]^3−/4−^ electro-oxidation, large cathode peak potentials (E_c_) of [Fe(CN)_6_]^3−/4−^ electrical recovery, and a smaller potential difference between E_c_ and E_a_ (∆E) are observed on all phyto-AuNPs/GCE. It should be noted that ∆E decreases in the GCE—gb-AuNPs/GCE–bc-AuNPs/GCE–sb-AuNPs/GCE series. For sb-AuNPs/GCE ∆E is 200 mV less than for GCE. The anode and cathode current peaks on cyclic voltammograms of [Fe(CN)_6_]^3−/4−^ electrical conversion grow on phyto-AuNPs/GCE as compared with GCE, while a more noticeable increase is observed for the anode current peak. The ratio of anode current peaks to cathode current peaks (I_a_/I_c_) is greater for phyto-AuNPs/GCE than for GCE. Sb-AuNPs/GCE has the highest value, almost equal to 1. Thus, the reversibility of [Fe(CN)_6_]^3−/4−^ electrical conversion, characterized by ∆E and I_a_/I_c_, improves on phyto-AuNPs/GCE as compared with GCE and with a decrease in the size of phyto-AuNPs (Table 4).

A similar less peak separation for [Fe(CN)_6_]^3−/4−^ with a decreasing size of gold nanoparticles was established by Kalimuthu and John [47]. The observed pattern is explained by faster electron transfer kinetics for small AuNPs and rather slow electron transfer kinetics for larger nanoparticles. The existence of a correlation between the thermodynamic properties of the surface and the kinetics of the electrode reaction is also proved by the results obtained by Brainina et al. who stated a linear relationship between the Gibbs free surface energy that increases with a decreasing nanoparticle size, and the rate of the electrode reaction [25].

### 3.6. Analytical Application of sb-AuNPs

**Figure 9:**
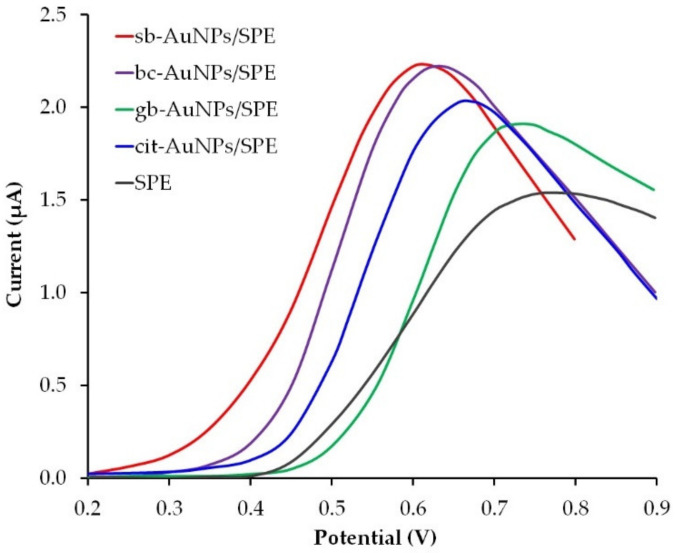
LS voltammograms of 0.1 mM UA on different electrodes in PBS pH 5. Potential scan rate 0.05 V s^−1^. Synthesis conditions: C(HAuCl_4_) = 1 mM, V_extr_ = 0.75 mL (for sb-AuNPs), V_extr_ = 1.0 mL (for bc-AuNPs and gb-AuNPs), and pH of all extracts: 12.

Sweat contains various biomolecules which makes it a diagnostic fluid for non-invasive monitoring of the human physiological state. For example, ascorbic (AA) and uric acids (UA) can be found in the dermis as well as in the epidermis, and are secreted with sweat. AA is involved in a variety of metabolic skin processes, for example, the formation of collagen. On the other hand, vitamin C deficiency can cause or aggravate the occurrence and development of such skin diseases as atopic dermatitis and porphyria cutanea tarda [48]. UA contributes to wound healing processes. Its role as an agent of gout has been well studied and is associated with a higher level of UA not only in blood, but in sweat as well [49,50]. Both AA and UA belong to non-enzymatic antioxidants and contribute to the regulation of redox homeostasis and decontamination of the skin from reactive oxygen species, therefore, the design of skin-attachable (epidermal) analytical devices that are capable to detect UA and AA in sweat secretions sensitively and selectively is of great concern. Electrochemical sensors are characterized by simplicity, sensitivity, selectivity, the possibility of miniaturization, and can be successfully used for in situ skin analysis of various biomarkers for point-of-care diagnostics. Several studies reported on the development of epidermal electrochemical sensors for the detection of UA [50] and AA [51] acids in human sweat. These sensors do not contain biocompatible nanoparticles in the surface layer. In our opinion, the immobilization of phytosynthesized gold nanoparticles on the surface of the working electrode could benefit the sensor biocompatibility and its sensory properties. To evaluate the analytical capabilities of phyto-AuNPs in the detection of uric acid, a screen-printed electrode (SPE) was used, since its planar design ensures the closest contact with the skin. Gold nanoparticles synthesized with the use of alkaline plant extracts (pH 12) were immobilized by the drip method on the SPE surface. Figure 9 illustrates LS voltammograms of UA on empty SPE and SPE modified with phyto-AuNPs that were phytosynthesized with the extracts of strawberry, gooseberry, and black currant. There is a decrease in the peak potential of UA oxidation from an empty SPE to sb-AuNPs/SPE, therefore, in further studies, the most electrochemically active sb-AuNPs/SPE was used. It was compared with the SPE modified with widely used gold nanoparticles obtained by the Turkevich citrate method (cit-AuNPs). As can be seen from Figure 9, the peak potential of UA electro-oxidation occurs earlier at sb-AuNPs/SPE than at cit-AuNPs/SPE, which indicates greater electrochemical activity of sb-AuNPs/SPE.

Derivative anodic voltammograms of UA and corresponding calibration curves di/dE = f(C), obtained with the help of sb-AuNPs/SPE and cit-AuNPs/SPE, are given in Figure S1 in the Supplementary Materials. The calibration graphs of UA at sb-AuNPs/SPE are linear in two concentration ranges of 0.1−0.98 μM and 0.98−190 μM with linear equations as di/dE(μA/V) = 0.038 + 0.617C_UA_ (μM), R^2^ = 0.993 and di/dE(μA/V) = 0.602 + 0.169C_UA_(μM), R^2^ = 0.996. The calibration graphs of UA at cit-AuNPs/SPE are linear in the concentration range of 0.2–190 μM with linear equations as di/dE(μA/V) = 0.342 + 0.128C_UA_ (μM), R^2^ = 0.992. These data indicate a higher sensitivity of sb-AuNPs/SPE to low concentrations of UA as compared with cit-AuNPs/SPE. A comparison of the analytical characteristics of the electrodes modified with sb-AuNPs and cit-AuNPs and used to detect UA and AA under model conditions is presented in Table 5. The data related to the determination of ascorbic acid with the use of the carbon veil electrode (CVE), modified with gold nanoparticles, were taken from our earlier work [19]. Table 5 shows that the electrodes modified with sb-AuNPs have better analytical characteristics as compared with the electrodes modified with cit-AuNPs. The linear ranges for UA and AA are 0.1–190 µM and 1–5750 µM, respectively, which makes it possible to measure these analytes at the level of their physiological content in human sweat (10–90 µM for UA [50] and 10–50 µM for AA [22]).

## 4. Conclusions

Eco-friendly synthesis of nanoparticles with the help of plant extracts and the use of these phytonanoparticles in various fields of science and technology is one of the newest and promising fields of research. Despite certain recent achievements in phytosynthesis, there is a research problem caused by the lack of knowledge about electrochemical properties of phytonanoparticles, which hinders their purposeful and effective use as modifiers of electrochemical interfaces. The present work aimed at studying electrochemical properties of gold nanoparticles that were synthesized by a hydrothermal method with the use of extracts from strawberry, black currant, and gooseberry leaves that possess bioreducing and stabilizing properties. The impact of the variable conditions of phytosynthesis (aliquot, AOA, and pH of plant extracts) on the parameters of electro-oxidation (potential and peak current) of phytosynthesized gold nanoparticles were discussed. It was found that an increase in aliquot, AOA, and pH of the plant extract contributes to the production of smaller and electrochemically active phyto-AuNPs. The research indicates that with a decrease in the size of nanoparticles, the reversibility of the electrical conversion of [Fe(CN)_6_]^3−/4−^ improves in the gb-AuNPs/GCE–bc-AuNPs/GCE–sb-AuNPs/GCE series. It was shown that the electrodes modified with photosynthesized sb-AuNPs have better analytical characteristics in the determination of uric and ascorbic acids than the electrodes modified with cit-AuNPs synthesized by the widely used Turkevich method. The obtained results indicate good prospects for using biocompatible phytosynthesized gold nanoparticles in epidermal electrosensing of biomarkers for point-of-care diagnostics.

## Figures and Tables

**Table 1:** The impact of washing gold sols synthesized with 0.75 mL of plant extracts (pH 6) on phyto-AuNPs electro-oxidation parameters.

Phyto-AuNPs	Peak Potential, V		Peak Current, μA	
	Without Purification	After Purification	Without Purification	After Purification
sb-AuNPs	0.85 ± 0.01	0.85 ± 0.02	7.3 ± 0.9	31.3 ± 3.0
bc-AuNPs	0.95 ± 0.03	0.94 ± 0.02	4.4 ± 0.6	18.8 ± 3.0
gb-AuNPs	1.00 ± 0.05	0.95 ± 0.03	2.0 ± 0.1	8.7 ± 0.7

**Table 2:** Parameters of electrochemical impedance for GCE and sb-AuNPs/GCEs using a Randles equivalent cell.

Electrode	V_extr_ Used in the sb-AuNPs Synthesis, mL	R_s_, Ω	R_ct_, kΩ	Q, μMho	n	W, μMho
GCE	0	99.0 ± 4.6	0.87 ± 0.11	10.2 ± 1.1	0.65 ± 0.03	148 ± 9
sb-AuNPs/GCE	0.25	95.5 ± 4.5	1.60 ± 0.20	4.7 ± 0.6	0.71 ± 0.02	160 ± 13
	0.50	111.0 ± 5.0	3.29 ± 0.21	21.2 ± 2.1	0.77 ± 0.05	176 ± 14
	0.75	105.7 ± 9.4	3.44 ± 0.18	18.7 ± 2.2	0.63 ± 0.04	158 ± 14
	1.0	95 ± 12	8.4 ± 1.2	7.2 ± 1.5	0.66 ± 0.10	182 ± 13

R_s_—solution resistance of the electrolyte; R_ct_—charge transfer resistance; Q—constant phase element; n—empirical constant reflecting electrode surface heterogeneity; and W—Warburg element.

**Table 3:** Characteristics of sb-AuNPs obtained by UV–vis and DLS. (Synthesis: 5.0 mL of 1 mM HAuCl_4_ + 0.75 mL of strawberry leaf extract with different pH).

pH of Strawberry Leaf Extract	DLS		UV–Vis
	ζ-Potential, mV	d_hd_, nm	d, nm
3	−28 ± 1	38 ± 1	12 ± 1
6	−27 ± 1	30 ± 1	10 ± 1
12	−42 ± 4	23 ± 1	6 ± 1

ζ-potential—electrokinetic zeta-potential; d_hd_—hydrodynamic diameter; and d—diameter derived from Equation (2).

**Table 4:** Parameters of electrochemical transformation of 5.0 mM [Fe(CN)_6_]^3−/4−^ in 0.1 M KCl on GCE and phyto-AuNPs/GCE. Conditions of phyto-AuNPs synthesis: C(HAuCl_4_) = 1 mM, V_extr_ = 0.75 mL (for sb-AuNPs), V_extr_ = 1.0 mL (for bc-AuNPs and gb-AuNPs), and pH of all extracts: 12.

Electrode	d, nm	E_a_, V	E_c_, V	∆E, V	I_a_, μA	I_c_, μA	I_a_/I_c_
sb-AuNPs/GCE	6 ± 1	0.46 ± 0.04	−0.13 ± 0.02	0.59 ± 0.02	23.9 ± 0.2	−23.4 ± 0.2	1.0 ± 0.1
bc-AuNPs/GCE	6 ± 1	0.52 ± 0.05	−0.12 ± 0.02	0.64 ± 0.02	21.6 ± 0.8	−22.7 ± 0.7	1.0 ± 0.2
gb-AuNPs/GCE	9 ± 1	0.59 ± 0.01	−0.18 ± 0.01	0.77 ± 0.03	19.2 ± 0.1	−21.6 ± 0.4	0.9 ± 0.1
GCE	-	0.63 ± 0.13	−0.16 ± 0.05	0.79 ± 0.17	18.4 ± 0.1	−21.3 ± 0.4	0.8 ± 0.1

d—diameter derived from Equation (2); E_a_—anode peak potential; E_c_—cathode peak potential; ∆E—anode and cathode potential difference; I_a_—anode peak current; I_c_—cathode peak current; and I_a_/I_c_—ratio of anode to cathode peak currents.

**Table 5:** A comparison of the analytical characteristics of the electrodes modified with sb-AuNPs and cit-AuNPs and used to detect UA and AA under model conditions by LSV.

Parameter	Uric Acid		Ascorbic Acid [19]	
	sb-AuNPs/SPE	cit-AuNPs/SPE	Phyto-AuNPs/CVE	cit-AuNPs/CVE
Limit of detection, μM	0.16	1.03	0.05	0.20
Limit of quantification, μM	0.49	3.13	0.15	0.60
Linear range, μM	0.1−0.98, 0.98−190	0.2–190	1–10, 10–5750	1–10, 10–11,700
Sensitivity, μA/μM	0.617, 0.169	0.128	0.130, 0.050	0.077, 0.025
S_r_ of response of minimal concentration, %	3	4	1.4	3.6

## Data Availability

Not applicable.

## Supplementary Materials

The following supporting information can be downloaded at: https://www.mdpi.com/article/10.3390/s22010311/s1, Table S1: Characterization of phyto-AuNPs by UV–Vis spectrophotometry, Table S2: AOA of aqueous solutions containing different aliquots of plant extracts and at different pH, Figure S1: Derivative anodic voltammograms with increasing concentrations of UA (0.1–190 μM) at the sb-AuNPs/SPE (a). Corresponding calibration curves di/dE = f(C_UA_). Inset: lowest linear range (b). Derivative anodic voltammograms with increasing concentrations of UA (0.2–190 μM) at the cit-AuNPs/SPE (c). Corresponding calibration curve di/dE = f(C_UA_) (d). Background: PBS (pH 5), ν = 0.05 V s^−1^.
