# Supplementary material for: Electrochemical Properties of Phytosynthesized Gold Nanoparticles for Electrosensing
Source: Sensors (Basel). 2021 Dec 31;22(1):311. doi: 10.3390/s22010311 (PMC8749774; doi:10.3390/s22010311)
Supplement: Supplementary file 1 [file sensors-22-00311-s001.zip › sensors-1494443-supplementary.pdf]

sensors

Supplementary Materials

Electrochemical Properties of Phytosynthesized Gold Nanoparticles for Electrosensing

Natalia Yu. Stozhko [1,*], Maria A. Bukharinova [2], Ekaterina I. Khamzina [1,2] and Aleksey V. Tarasov [2]

[1] Department of Physics and Chemistry, Ural State University of Economics, 8 Marta St., 62, 620144 Yekaterinburg, Russia
[2] Scientific and Innovation Center of Sensor Technologies, Ural State University of Economics, 8 Marta St., 62, 620144 Yekaterinburg, Russia; m.a.buharinova@usue.ru (M.A.B.); xei260296@mail.ru (E.I.K.); tarasov_a.v@bk.ru (A.V.T.)
* Correspondence: sny@usue.ru (N.Y.S.)

Table S1. Characterization of phyto-AuNPs by UV-Vis spectrophotometry.

Plant leaves used	Aliquot of the extract used in synthesis, ml	Characteristics of gold nanosuspension	
		d_{AuNPs}, nm	N_{AuNPs}, L^{-1}
Gooseberry (*Ribes uva-crispa*)	0.25	–	–
	0.50	34	$3.50×10^{12}$
	0.75	29	$7.80×10^{12}$
	1.00	25	$1.94×10^{13}$
Black currant (*Ribes nigrum*)	0.25	20	$2.38×10^{13}$
	0.50	15	$1.34×10^{14}$
	0.75	13	$2.83×10^{14}$
	1.00	11	$5.64×10^{14}$
Strawberry (*Fragaria vesca*)	0.25	18	$5.34×10^{13}$
	0.50	14	$1.98×10^{14}$
	0.75	12	$4.74×10^{14}$
	1.00	10	$8.37×10^{14}$

d_{AuNPs} and N_{AuNPs}–diameter and numerical concentration of gold nanoparticles, respectively.

Table S2. AOA of aqueous solutions containing different aliquots of plant extracts and at different pH.

Plant	pH	Extract aliquot, mL	Plant extract AOA, mM-eq
Gooseberry (*Ribes uva-crispa*)	3	1.00	0.34 ± 0.02
	6	0.50	0.14 ± 0.01
	6	0.75	0.24 ± 0.02
	6	1.00	0.29 ± 0.02
	12	1.00	0.26 ± 0.02
Black currant (*Ribes nigrum*)	3	1.00	0.72 ± 0.05
	6	0.25	0.23 ± 0.02
	6	0.50	0.43 ± 0.03
	6	0.75	0.59 ± 0.04
	6	1.00	0.73 ± 0.06
	12	1.00	0.51 ± 0.03
Strawberry (*Fragaria vesca*)	3	0.75	1.55 ± 0.08
	3	1.00	1.99 ± 0.09
	6	0.25	0.63 ± 0.05
	6	0.50	1.21 ± 0.09
	6	0.75	1.57 ± 0.11
	6	1.00	2.16 ± 0.12
	12	0.75	1.72 ± 0.10
	12	1.00	2.20 ± 0.12

AOA–antioxidant activity.

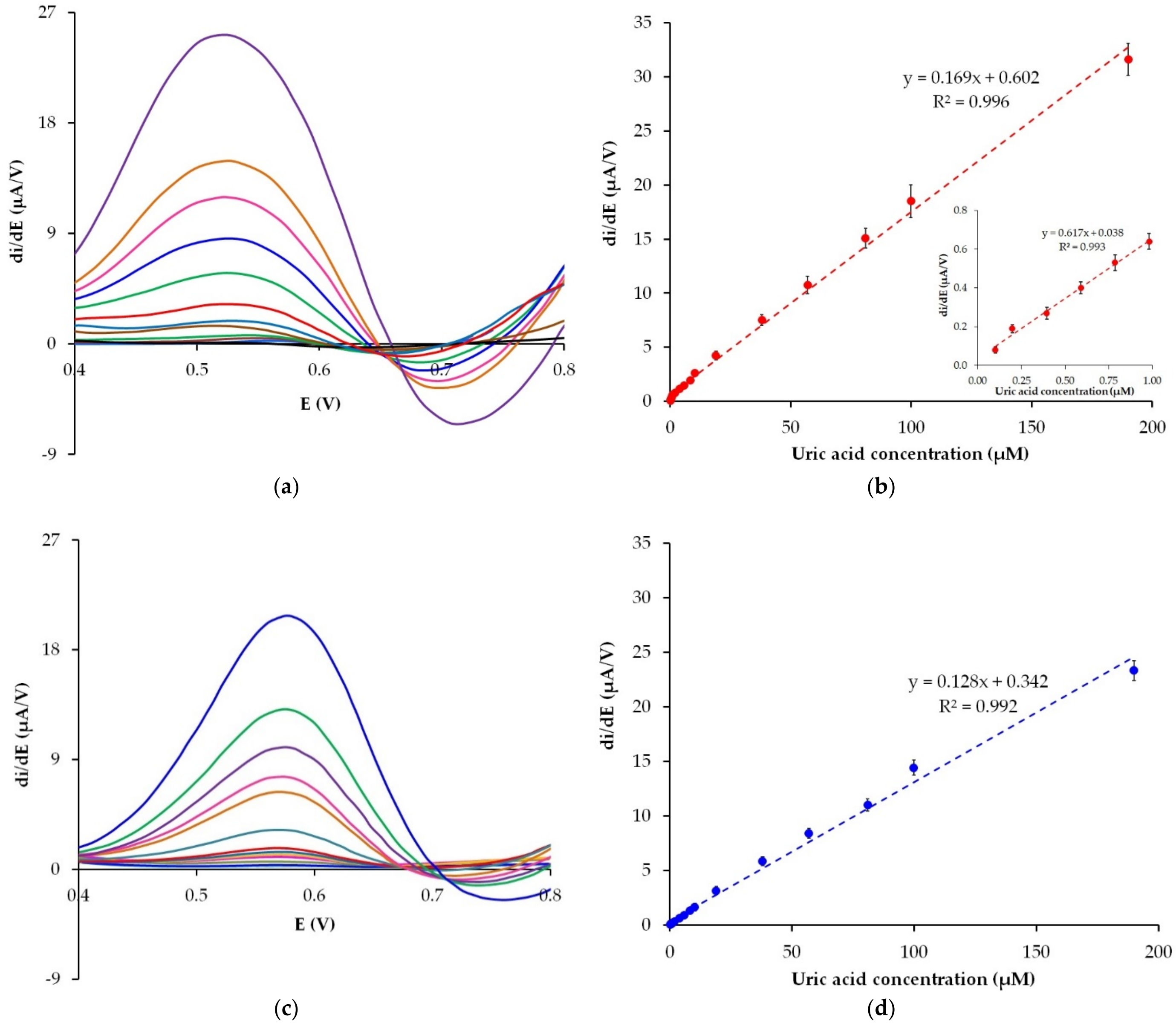

Figure S1. Derivative anodic voltammograms with increasing concentrations of UA (0.1–190 µM) at the sb-AuNPs/SPE (**a**). Corresponding calibration curves di/dE = f(C_{UA}). Inset: lowest linear range (**b**). Derivative anodic voltammograms with increasing concentrations of UA (0.2–190 µM) at the cit-AuNPs/SPE (**c**). Corresponding calibration curve di/dE = f(C_{UA}) (**d**). Background: PBS (pH 5), ν=0.05 V s^{-1}. UA–uric acid.
